# Direct Measurement of Performance: A New Era in Antimicrobial Stewardship

**DOI:** 10.3390/antibiotics8030127

**Published:** 2019-08-24

**Authors:** Majdi N. Al-Hasan, Hana Rac Winders, P. Brandon Bookstaver, Julie Ann Justo

**Affiliations:** 1 School of Medicine, University of South Carolina, Columbia, SC 29209, USA; 2 Department of Medicine, Division of Infectious Diseases, Palmetto Health University of South Carolina Medical Group, Columbia, SC 29203, USA; 3 Department of Clinical Pharmacy and Outcomes Sciences, University of South Carolina College of Pharmacy, Columbia, SC 29208, USA; 4 Department of Pharmacy, Prisma Health Richland, Columbia, SC 29203, USA

**Keywords:** Antibiotics, resistance, broad-spectrum agents, hospital epidemiology, antibiotic utilization, infection control, infection prevention, *Pseudomonas aeruginosa*, *Acinetobacter baumannii*, extended-spectrum beta-lactamases, carbapenem-resistant *Enterobacteriaceae*, methicillin-resistant *Staphylococcus aureus*

## Abstract

For decades, the performance of antimicrobial stewardship programs (ASPs) has been measured by incidence rates of hospital-onset *Clostridioides difficile* and other infections due to multidrug-resistant bacteria. However, these represent indirect and nonspecific ASP metrics. They are often confounded by factors beyond an ASP’s control, such as changes in diagnostic testing methods or algorithms and the potential of patient-to-patient transmission. Whereas these metrics remain useful for global assessment of healthcare systems, antimicrobial use represents a direct metric that separates the performance of an ASP from other safety and quality teams within an institution. The evolution of electronic medical records and healthcare informatics has made measurements of antimicrobial use a reality. The US Centers for Disease Control and Prevention’s initiative for reporting antimicrobial use and standardized antimicrobial administration ratio in hospitals is highly welcomed. Ultimately, ASPs should be evaluated based on what they do best and what they can control, that is, antimicrobial use within their own institution. This narrative review critically appraises existing stewardship metrics and advocates for adopting antimicrobial use as the primary performance measure. It proposes novel formulas to adjust antimicrobial use based on quality of care and microbiological burden at each institution to allow for meaningful inter-network and inter-facility comparisons.

## 1. Introduction: Importance of Antimicrobial Stewardship Metrics

It is imperative for the success of any antimicrobial stewardship program (ASP) to have objective measures for performance evaluation. Direct measurement of ASP performance via process measures (e.g., antimicrobial use) and/or outcome measures (e.g., *Clostridioides difficile* infection [CDI]) is currently recommended by clinical guidelines to improve quality care and prevent antimicrobial resistance [1]. This process ensures that both hospital administration and ASP team members have consistent goals and expectations. It provides ASPs with the opportunity to periodically self-reflect on their performance and discuss long-term planning to achieve their aims. This also creates national and local standards to compare ASPs in different healthcare systems after adjustments for potential differences across institutions [1].

ASP metrics are often categorized by type into antimicrobial use (AU) measures, process measures, quality measures, costs and clinical outcome measures. Expert panels assembled among adult and pediatric stewards were challenged to develop a set of metrics perceived as both useful and logistically feasible for adoption by ASPs as performance metrics [2,3]. Variability in practice areas, institutions, resources and infrastructure all impede the utility of many proposed ASP metrics. The true impact of an ASP on quality and clinical outcome measures specifically is also debatable, given the patient complexities and confounders present. Do these metrics actually measure ASP “performance” or “value” or “efficiency”, a combination of these factors, or none at all?

While several quantitative measures (e.g., antimicrobial use and costs) are often considered frontline metrics and central to ASP operations, noted expert stewards have proposed a shift in focus to quality and patient outcomes to demonstrate enhanced program value [4,5,6]. Many regulatory and quality improvement organizations (e.g., Agency for Healthcare Research and Quality) have established infectious diseases metrics designed to measure quality which are often tied to reimbursement [4]. The changing landscape of reimbursement in the US healthcare system and growing transparency of quality and safety measures through public reporting will likely impact ASPs and potentially influence key metrics tied to performance evaluation. Collaboration between ASPs, healthcare administration and quality divisions is imperative in order to maintain consistency in measured success. In this narrative review, we discuss the landscape of proposed ASP metrics and compare their value and utility as measures of ASP performance focusing on acute care hospitals.

## 2. Dynamics of Antimicrobial Stewardship and Infection Prevention and Control Programs 

The re-emergence of CDI as a significant threat in the early 2000s was arguably the single most important factor increasing awareness of risks associated with antimicrobials at both the public and institutional level. The potential of patient-to-patient transmission of *C. difficile* spores make clusters or outbreaks of CDI an imminent threat to hospitals. CDI created a common target for both Infection Prevention and Control Programs and ASPs and facilitated a dynamic relationship between both teams. Contact isolation of hospitalized patients with CDI and hand hygiene of healthcare workers with soap and water have been the cornerstone of Infection Prevention and Control Program efforts to reduce transmission of *C. difficile* spores within the hospital. At the same time, reduction in unnecessary use of broad-spectrum antimicrobials may reduce the risk of hospital-onset CDI (HO-CDI). For this reason, many Infection Prevention and Control Programs started monitoring antimicrobial use in the hospital to enhance their CDI interventions, often utilizing the same team members. On other occasions, ASPs emerged under the Infection Prevention and Control Program umbrella. It was convenient to use the incidence rate of HO-CDI as a metric for both Infection Prevention and Control Programs and ASPs. The requirement for hospitals within the United States to publicly report the incidence rate of HO-CDI through National Healthcare Safety Network (NHSN) and paucity of other measures of ASP performance only emphasized this existing concept.

Similarly, institutional Infection Prevention and Control Programs have been monitoring and intervening to prevent patient-to-patient transmission of multidrug-resistant (MDR) bacteria: initially, extended-spectrum beta-lactamase-producing Enterobacteriaceae (ESBLE) and methicillin-resistant *Staphylococcus aureus* (MRSA), then carbapenem-resistant Enterobacteriaceae (CRE). Since antimicrobial use predisposes to colonization and infections with MDR bacteria [7,8], incidence rates of infections or colonization with MDR bacteria were often used as a quality measure of ASP performance. Mandatory reporting of these infections added another layer of convenience.

Over time, ASPs have evolved and have become more focused on quality of patient care, including optimization of antimicrobial management. At the same time, *C. difficile*, MRSA and ESBLE have emerged as community-onset pathogens rather than predominant causes of nosocomial infections [9,10,11,12]. Antimicrobial resistance of predominantly hospital-onset bacteria, such as *Pseudomonas aeruginosa* and *Acinetobacter baumannii*, has become the most imminent threat to hospitals in the US [13].

Moreover, with the emergence of community-acquired MRSA, intravenous vancomycin has become the most commonly used antimicrobial in US hospitals. The increasing use of vancomycin, by itself or in combination with piperacillin/tazobactam, has contributed to an increase in antimicrobial-associated nephrotoxicity in hospitalized patients [14,15]. Monitoring the use of nephrotoxic antimicrobial agents has been added to the daily duties of ASPs. This shifting focus has allowed a greater degree of independence from Infection Prevention and Control Programs and has made it difficult to use the same personnel for both Infection Prevention and Control Programs and ASPs.

## 3. Comparison of Various Antimicrobial Stewardship Metrics

### 3.1. Clostridioides difficile Infection

Prevention of CDI is one of the main benefits of antimicrobial stewardship. Current or prior antimicrobial use contributes to CDI due to changes in intestinal microbiota and decreased competition against *C. difficile* [16]. Therefore, it is intuitive to use CDI as a measure of ASP performance in hospitals [1,2]. However, the multifactorial nature of CDI, possibility of person-to-person transmission irrespective of antimicrobial use and site of acquisition, and changing incidence rate of CDI based on diagnostic testing methods or algorithms, argue against its use as the primary ASP metric.

#### 3.1.1. CDI Diagnosis

CDI can only be diagnosed based on laboratory testing. Using a highly sensitive test, such as PCR, would increase the incidence of CDI compared to toxin A/B antibody–antigen assays [17]. An increase in the incidence rate of HO-CDI was observed when switching from toxin A/B antibody–antigen testing to PCR [18]. The use of diagnostic tests with varying sensitivities makes comparison of HO-CDI incidence rates across hospitals impractical. A recent study demonstrated that only 20% of in-hospital testing for CDI was appropriate [19]. Inappropriate testing for CDI resulted in overtreatment and inaccurate publicly reported metrics [19]. Even when the same laboratory diagnostic test is used, a change in institutional policy or algorithm for CDI testing influenced CDI incidence rates. An institutional requirement for testing all hospitalized patients with liquid stools was associated with higher incidence rate of HO-CDI in Scotland [20]. *C. difficile* PCR does not differentiate colonization from infection. Given the large proportion of inappropriate CDI testing in hospitals, a clinical decision-making tool to improve the appropriateness of testing likely has a much higher impact in reducing HO-CDI rates than any ASP intervention. This confounding makes it difficult to correlate incidence of HO-CDI with ASP activities aiming to optimize antimicrobial use. Instead, this argues for design of a diagnostic stewardship metric in collaboration with microbiology, central laboratory and Infection Prevention and Control Programs.

#### 3.1.2. Relatively Low Incidence of CDI

Most clinical studies of CDI adopt a case-control design due to the relative infrequency of CDI in hospitalized patients. In two cohorts, only 2–4% of hospitalized patients with gram-negative bloodstream infections developed CDI, despite receipt of broad-spectrum antimicrobial therapy [21,22]. Although still considered a major risk, the relatively large number needed to harm constitutes a challenge for ASPs attempting to demonstrate effectiveness of their interventions. Based on such data, ASPs are required to streamline or discontinue 25–50 courses of broad-spectrum antimicrobials to potentially prevent one case of CDI. Discontinuation of antimicrobial therapy is one of the most impactful outcomes of any ASP intervention; however, it is a much less frequent intervention than de-escalation of antimicrobial therapy or reduction in proposed treatment duration [23]. Designing an ASP intervention to reduce the incidence rate of CDI requires a tremendous amount of time, resources and dedication. This is likely the reason for the relatively small number of published studies demonstrating successful reduction in CDI via ASP interventions, despite several decades of focus in this area. To our knowledge, only early de-escalation of broad-spectrum antimicrobial therapy (within 48 h) has been associated with a reduction in CDI risk [22]. Early de-escalation of antimicrobial therapy requires robust ASP, rapid diagnostics, timely electronic alerts, and experienced personnel to act on these alerts. Conventional de-escalation of antimicrobial therapy (after 4 days) has demonstrated non-inferiority to broad-spectrum therapy, but is yet to show a significant reduction in CDI [24].

#### 3.1.3. Multifactorial Etiology of CDI

In addition to antimicrobials, many other independent risk factors have been associated with development of CDI, such as chemotherapy and proton-pump inhibitors [25]. Moreover, potential for person-to-person transmission of *C. difficile* spores makes Infection Prevention and Control Program efforts far more important than those of ASPs in reducing the incidence of HO-CDI. Given the large number of ASP interventions required to prevent CDI, a cluster of CDI in one unit of the hospital may cancel out an entire year’s worth of ASP efforts. Another factor that has not been widely studied is the impact of antimicrobials used prior to hospital admission on the risk of HO-CDI. Hospital admissions secondary to community-onset CDI continue to rise and are not as intensely monitored by Infection Prevention and Control Programs or ASPs, but may also pose similar risk to institutional outbreaks. Most institutional ASPs have no control over antimicrobials received in ambulatory settings or other hospitals prior to referral. 

#### 3.1.4. Difficulty of Designing a Successful ASP Intervention for CDI

Although the association between antimicrobial use and CDI is strong, it remains controversial which antimicrobial agents/classes are more likely to contribute to CDI [26]. There is general agreement that the broader the spectrum of antimicrobials, the higher the risk of CDI; however, there are notable exceptions to this rule, such as clindamycin [27]. Interpretation of the literature is difficult, due to use of different definitions and inconsistent methodology. To increase sample size and power, community-onset and HO-CDI were merged despite vast differences in the spectrum of activity of oral and intravenous antimicrobials used in the two respective settings [28]. Moreover, all penicillins were classified in one category, despite the huge difference in the spectrum of activity of piperacillin-tazobactam and penicillin G, for example [29]. Even the well-designed interventions which have reported a reduction in the incidence rate of CDI after antimicrobial formulary changes did not assess the collateral damage of the intervention on antimicrobial resistance [20,30]. It is worrisome that some formulary restrictions designed for reducing CDI risk may encourage the use of antipseudomonal beta-lactams and carbapenems [30]. This contradicts recent large cohorts demonstrating the highest odds of CDI among hospitalized patients receiving antipseudomonal beta-lactams [22,31]. In addition, the linear increase in antimicrobial resistance of *E. coli* and other Enterobacteriaceae bloodstream isolates to aminopenicillins and first-generation cephalosporins limits de-escalation options from antipseudomonal beta-lactams to intravenous ceftriaxone or oral fluoroquinolones on many occasions [22,32,33]. The long-term consequences of increasing antimicrobial resistance rates to antipseudomonal beta-lactams and carbapenems secondary to excessive use may exceed any potential early benefits from this strategy [34,35]. A subtle decline in CDI at the expense of increasing antimicrobial resistance rates of already difficult to treat bacteria, such as *P. aeruginosa* and *A. baumannii*, constitutes one step forward and two steps back for the longevity of the ASP and the institution.

### 3.2. Incidence Rates of Infections or Colonization with MDR Bacteria

#### 3.2.1. Extended-Spectrum Beta-Lactamase-Producing *Enterobacteriaceae* (ESBLE)

Controlling outbreaks and reducing transmission of ESBLE in the hospital setting have been common goals for both Infection Prevention and Control Programs and ASPs since this resistance mechanism was first described in 1983 [36]. This is conceivable since exposure to antimicrobials, particularly beta-lactams and fluoroquinolones, is a risk factor for infection or colonization with ESBLE [7,8]. Moreover, ESBLE may be transmitted from person-to-person within hospitals or other settings. At the turn of the century, ESBLE emerged as community-onset bacteria likely due to availability and widespread use of broad-spectrum oral antimicrobials in the community, such as extended-spectrum cephalosporins and fluoroquinolones [11,37]. The incidence rate of hospital-acquired ESBLE infections have remained relatively stable over the past decade, due to effective Infection Prevention and Control Programs and ASPs [12]. On the other hand, the lack of such programs in ambulatory settings and long-term care facilities has contributed to an increase in the incidence rate of community-onset ESBLE infections [12]. It is estimated that 80% of ESBLE infections in the US are acquired outside the hospital [8,12].

Another limitation of using ESBLE as a measure of ASP performance is the lag between ESBLE colonization and infection. Colonization with ESBLE within the past one year has been associated with increased risk of ESBLE infections [8,38]. Patients may be colonized with ESBLE due to antimicrobial use in the community. If a urinary culture is obtained on the fourth day of hospitalization for appropriate or inappropriate indications, the ESBLE isolate will be classified as nosocomial, even in the absence of any antimicrobial use during the index hospitalization [39]. This limits the utility of ESBLE as a measure of ASP performance.

#### 3.2.2. Methicillin-Resistant *Staphylococcus aureus* (MRSA)

The emergence of MRSA as community-acquired bacteria by the end of last century makes the incidence of hospital-onset MRSA infections or colonization a less useful ASP metric. There is a suggestion that antimicrobial use may predispose to MRSA colonization or infection. A recent study demonstrated that the restriction of fluoroquinolone and macrolide use, among other antimicrobials, was associated with a reduction in MRSA infection rates [40]. However, this association was temporal, at best, and a decline in MRSA infection/colonization rates was demonstrated elsewhere without formulary changes [41,42,43]. In the era of increasing antimicrobial resistance rates, class restrictions of already limited antimicrobial treatment options for hospitalized patients with serious infections seem counterproductive. Given the high prevalence of community-acquired MRSA strains and widespread use of fluoroquinolones in the community, it is unrealistic to expect formulary restrictions of fluoroquinolones in hospitals to impact overall MRSA rates. Restricting fluoroquinolone use in the community to specific indications, such as acute pyelonephritis and community-onset pneumonia, seems more reasonable [44].

#### 3.2.3. Carbapenem-Resistant *Enterobacteriaceae* (CRE)

Carbapenem exposure is a risk factor for CRE infections or colonization [45]. Since carbapenems are currently only available in intravenous form in the US, the incidence rate of CRE appears more relevant to institutional ASPs than that of ESBLE and MRSA. The potential for person-to-person transmission have made long-term care facilities reservoirs for CRE, likely due to lack of effective Infection Prevention and Control Programs and ASPs. The lag between CRE colonization and infection, as well as the potential for receiving carbapenems at other facilities, pose some limitations to using CRE incidence rates to evaluate ASP performance. The potential availability of oral carbapenems in the US in the near future may change the epidemiology of CRE infections, in an unfortunate repeat of the community-onset ESBLE phenomenon.

#### 3.2.4. Antimicrobial-Resistant *P. aeruginosa* and *A. baumannii*

*P. aeruginosa* and *A. baumannii* are predominantly hospital-onset pathogens [46]. They are ubiquitous bacteria which are difficult to eliminate from hospital environments. Hospitalized patients may become colonized with these bacteria due to either heavy exposure from prolonged hospitalization or antimicrobial selection pressure [47,48]. The presence of open wounds, mechanical ventilation, and urinary or central venous catheters place hospitalized patients at higher risk of infections with these bacteria [46,49,50]. Resistance to antipseudomonal beta-lactams and carbapenems among these isolates poses serious challenges to hospitals due to the lack of safe and effective antimicrobial treatment options [35]. Outbreaks of infections due to MDR *P. aeruginosa* and *A. baumannii* are devastating to both patients and hospitals, associated with high mortality rates and high costs of treatment. The amount of time, resources and personnel dedicated to the containment of such outbreaks is enormous, occasionally requiring unit closures and massive financial burdens [51,52]. For this reason, *P. aeruginosa* and *A. baumannii* are at the top of the World Health Organization global list of critical priority [13].

Inpatient antimicrobial use is by far the most important factor influencing antimicrobial resistance rates of these hospital-onset isolates [53,54,55,56,57]. The recent increase in utilization of antipseudomonal beta-lactams and carbapenems in US hospitals has been temporally associated with an increase in antimicrobial resistance rates of *P. aeruginosa* [34,35]. Using antimicrobial resistance of *P. aeruginosa* and *A. baumannii* as an ASP metric is logical and reasonable, but has limitations. First, changes in referral patterns may impact antimicrobial resistance at tertiary care centers. Second, nearly one-half of *P. aeruginosa* bloodstream isolates are acquired outside the hospital [48]. Community-onset *P. aeruginosa* infections are particularly common among immune compromised hosts and patients who received recent beta-lactams [48,49,50]. Antimicrobial resistance rates of strictly hospital-onset *P. aeruginosa* and *A. baumannii* isolates are a more equitable measure of ASP performance. This requires stratification by site of acquisition. If such stratification is not automated by clinical informatics, then resistance rates of hospital-onset isolates will have to be done manually. This is unlikely to be feasible for many ASPs, based on current resources in time and personnel.

### 3.3. Quality of Care

Quality of patient care is the most important antimicrobial stewardship principle [2,3,4,5]. The ultimate goal of ASPs is to optimize both empirical and definitive antimicrobial therapy for hospitalized patients with serious infections. 

#### 3.3.1. Appropriate Definitive Antimicrobial Therapy

Many ASPs utilize available healthcare informatics resources to identify suboptimal antimicrobial use among individual patient cases. The objective is to ensure patients who have positive clinical cultures, particularly from sterile sites, receive the most effective antimicrobial therapy. Appropriate therapy is not only a matter of receiving an antimicrobial agent with in vitro susceptibility against the microbial isolate. Rather, it is based on effectiveness as derived from large clinical studies and involves receiving an appropriately dosed agent based on the primary source of infection, the patient’s renal and hepatic function, and the minimal inhibitory concertation of the clinical isolate. The antimicrobial should also be administered via the appropriate route based on severity of infection, reliability of the enteral route, and bioavailability of the agent [58,59,60].

Since most currently available software for identification of bug–drug mismatches use in vitro susceptibility as a screening measure for appropriateness, many patients receiving inappropriate definitive therapy will not be identified. This includes patients receiving antimicrobial agents without activity at the site of the infection (e.g., daptomycin for MRSA pneumonia or nitrofurantoin for *E. coli* bloodstream infections). It would require a separate ASP intervention to review all cases of pneumonia or bloodstream infection to identify such cases. The variety of factors associated with the “appropriateness” of antimicrobial therapy makes it difficult to measure ASP performance based on this metric alone.

#### 3.3.2. Appropriate Empirical Antimicrobial Therapy

Receipt of appropriate empirical antimicrobial therapy is independently associated with survival and shorter duration of hospitalization in patients with serious bacterial infections [58,59,61,62,63]. Despite the excessive use of broad-spectrum agents in hospitals, up to 30% of patients still receive inappropriate empirical therapy [64]. As the focus of ASPs has shifted to quality, many ASPs have invested in measuring and improving the appropriateness of empirical therapy. The art is designing institutional management guidelines which increase the appropriateness of empirical therapy while also reducing overall use of broad-spectrum agents [65]. Institutional management guidelines based on local evidence, coupled with rapid microbial identification and vigorous ASP monitoring, have been demonstrated to successfully achieve both goals [66]. The proportion of patients receiving appropriate empirical antimicrobial therapy for serious infections is the most important measure for the quality of ASP performance. Many host factors, including age, comorbidities and acute severity of illness, impact both survival and hospital length of stay in patients with serious bacterial infections [58,63,67,68]. In reality, the only variable an ASP can directly control and modify is the selection of empirical antimicrobial therapy. In order to use appropriateness of empirical therapy as a metric for ASP performance, it would require identification of every patient with a particular clinical syndrome (e.g., bloodstream infections, sepsis, or pneumonia) or a representative random sample. Although this may be time-consuming and labor-intensive, many ASPs are currently monitoring the appropriateness of empirical therapy, particularly in patients with bloodstream infections [66,69,70,71,72].

### 3.4. Cost of Healthcare

Antimicrobial cost was, for the most part, the only specific measure of ASP performance. Nonetheless, an institutional antimicrobial budget may be influenced by changes in acquisition price or renegotiation of institutional contracts, as dictated by supply and demand in the market.

Cost reduction has historically been used to justify the presence of an ASP, including both the establishment of a new program and maintenance of an existing program. ASPs have demonstrated significant reductions in hospitals’ antimicrobial budgets, mostly by targeting and restricting unnecessary use of expensive antimicrobials (e.g., daptomycin, ceftaroline) [73,74,75,76]. However, cost savings generally plateau after few years. The cost of the acquisition of antimicrobials varies across institutions based on purchase volume and the ability of the healthcare system to negotiate a better deal. Occasional price hikes of commonly used agents make cost a less attractive metric. More importantly, the biggest cost savings institutional ASPs provide are hard to measure. These cost savings include reduction in length of hospital stay by improving the appropriateness of empirical antimicrobial therapy for serious infections [62,63]. Second, measurement of cost avoidance is also challenging. An ASP’s efforts in reducing antimicrobial resistance rates of hospital-onset bacteria minimizes the need for new and often expensive antimicrobials used for treatment of infections due to MDR bacteria (e.g., ceftolozane/tazobactam, ceftazidime/avibactam).

### 3.5. Antimicrobial Use

#### 3.5.1. Direct and Specific ASP Metric

Since ASPs manage antimicrobial therapy on a daily basis, harnessing antimicrobial use as the primary ASP metric is intuitive. Antimicrobial use represents the most direct measure of ASP performance. Most other metrics (e.g., CDI, MDR bacteria, and cost) provide indirect assessment of antimicrobial use. The assumption is that the higher the antimicrobial use within a hospital, the higher the HO-CDI and antimicrobial resistance. Contrary to incidence rates of HO-CDI and infections with MDR bacteria, institutional antimicrobial use is not affected by antimicrobials used outside the hospital. In addition, since the incidence of HO-CDI and infections with MDR bacteria may be reduced by Infection Prevention and Control Program efforts, antimicrobial use remains the only metric which differentiates the performance of ASPs from other patient safety and quality teams.

#### 3.5.2. Antimicrobial Use of Broad-Spectrum Agents

ASP priority should be given for measuring antimicrobial use of broad-spectrum agents, such as antipseudomonal beta-lactams and carbapenems. For long-term monitoring, it would be useful to measure collective antimicrobial use of all antipseudomonal beta-lactams (e.g., piperacillin/tazobactam, cefepime, meropenem). This would avoid fluctuations associated with a temporary shortage of one agent. Monitoring intravenous vancomycin use is also important given the potential risk of nephrotoxicity [14,15]. Monitoring aminoglycosides use is equally important, particularly in institutions with frequent use of aminoglycoside combination regimens. Measurement of antimicrobial use of commonly used agents for treatment of community-onset infections, such as third-generation cephalosporins and fluoroquinolones, is also useful to ensure antipseudomonal beta-lactams and carbapenems are not completely replaced by agents which are still associated with a high risk of CDI. It would be reasonable to monitor institutional antimicrobial use of all agents, resources permitting, in order to ensure a decline of overall antimicrobial use in the hospital. However, most ASPs would not be bothered if the decline in antimicrobial use of broad-spectrum agents was accompanied by an increase in antimicrobial use of narrower-spectrum agents (e.g., penicillin G, nafcillin, ampicillin/sulbactam, cefazolin). After all, this is reflective of their hard work in de-escalation of broad-spectrum antimicrobial therapy.

#### 3.5.3. Benefits of Reducing Antimicrobial Use 

A reduction in antimicrobial use of antipseudomonal beta-lactams and other broad-spectrum agents is possible through syndrome-specific and other ASP interventions [72]. In addition, monitoring antimicrobial use is also rewarding for an ASP as a decline in antimicrobial use of broad-spectrum agents may be observed as early as 6 months following a successful intervention [72,77,78]. In the long-term, reduction in antimicrobial use of broad-spectrum agents will result in a decline in HO-CDI and antimicrobial resistance assuming there are no major changes in the healthcare system (e.g., referral patterns, outpatient antimicrobial prescription rates, clusters of HO-CDI or MDR bacteria).

#### 3.5.4. Measurement of Antimicrobial Use

There are advantages and disadvantages of using days of therapy (DOT) or defined daily dose (DDD) as measures of antimicrobial use which are beyond the scope of this review. However, the goal of an ASP is to optimize, rather than minimize, antimicrobial therapy. Using DDD as a measure of antimicrobial use may punish ASPs for optimization of antimicrobial regimens in patients who truly need high doses of antimicrobials, such as patients with serious infections and augmented renal clearance. Conversely, institutions with relatively high rates of acute kidney injury due to heavy use of nephrotoxic agents may benefit from measuring DDD rather than DOT. Overall, DOT seems a fair indicator of performance for a local ASP.

An added benefit of antimicrobial use measurement is the frequent ability to stratify antimicrobial use by location and by time. This requires detailed knowledge of the denominator used in the generation of the antimicrobial use measurement, i.e., patient-days or the newer standard, days-present. Such data allows antimicrobial use to be locally compared across locations, such as hospital campus (in a multi-campus health system) or hospital unit, and by time, such as calendar month. This, in turn, helps ASPs identify areas within the institution where antimicrobial use appears excessive and helps design unit-specific or other targeted interventions.

## 4. Proposed Novel Antimicrobial Use (AU) Metrics

### 4.1. Adjustment of AU by Quality of Care

AU by itself is a measure of quantity, not quality of care. It would be valuable to provide reassurance that a reduction in AU of broad-spectrum agents is not achieved at the expense of appropriateness of therapy. Adjusting AU of broad-spectrum agents to the proportion of patients receiving appropriate empirical therapy incorporates quality of care and AU in one formula (Equation (1)):

(1) $$AU_{adjustedQ}=\frac{AU_{local}}{P_{appropriate}}$$

**Equation (1):** Antimicrobial use adjusted by quality of care as determined by appropriateness of empirical antimicrobial therapy.

Where *AU_adjustedQ_* is the adjusted AU by quality of care at an institution, *AU_local_* is the raw AU at a particular local institution; *P_appropriate_* is the proportion of patients receiving appropriate empirical antimicrobial therapy at that facility.

For example, using 100 DOT/1000 patient-days of antipseudomonal beta-lactams to provide appropriate empirical therapy to 90% of patients with gram-negative bloodstream infections at hospital A is better than using the same amount to cover only 80% appropriately at hospital B (100/0.9 = 111 vs. 100/0.8 = 125). This formula implies it would have taken 111 and 125 DOT/1000 patient-days, respectively, to provide appropriate empirical therapy to virtually all patients with this clinical syndrome at hospitals A and B, respectively (Table 1). This provides an assessment of the quantitative (AU) and qualitative (appropriateness of empirical antimicrobial therapy) performance of ASPs. It should be noted that antimicrobial resistance rates at an institution may influence the proportion of patients receiving appropriate empirical therapy.

Many ASPs are currently involved in management of bloodstream infections in order to optimize empirical therapy. The proportion of patients with bloodstream infections receiving appropriate empirical therapy is already available at such institutions [66,69,70,71]. Moreover, as institutions adopt new bundles for improvement of survival in patients with sepsis, it would be useful to measure the appropriateness of empirical antimicrobial therapy as part of this bundle. These bloodstream infection and sepsis cohorts may be used as representative samples for the adjustment of AU by the quality of care received at each institution.

### 4.2. Adjustment of AU by Institutional Microbiological Burden

Similar to any other ASP metric, comparisons of AU across institutions are not valuable without taking into account the differences in patient populations and microbiological burden at these facilities. For example, there is a wide variation in the incidence of *P. aeruginosa* among gram-negative bacteria at various institutions with higher incidence at tertiary care referral centers than community hospitals [79]. Since broad-spectrum antimicrobial agents are used to treat infections due to certain bacteria (e.g., antipseudomonal beta-lactams for *P. aeruginosa*), it is reasonable to adjust for the incidence of such isolates at a particular institution. Equation (2) can be used to adjust AU by institutional microbiological burden:

(2) $$AU_{adjustedM}=\frac{AU_{local}}{\left(\frac{I_{local}}{I_{overall}}\right)}$$

**Equation (2):** Antimicrobial use adjusted by microbiological burden at the institution.

Where *AU_adjustedM_* is the adjusted AU of antipseudomonal beta-lactams by microbiological burden at an institution, *AU_local_* is the raw AU of antipseudomonal beta-lactams at a particular local institution, *I_local_* is the incidence of the relevant organism(s) (i.e., *P. aeruginosa*) at that local institution, and *I_overall_* is its average incidence within the overall network or region.

As an example, *AU_local_* of antipseudomonal beta-lactams at hospitals A (tertiary care medical center) and B (rural community hospital) are both reported as 100 DOT/1000 patient-days. The *I_overall_* is 0.12 (12% of all gram-negative isolates within this network or region are *P. aeruginosa*, for instance). *I_local_* for hospitals A and B are 0.15 and 0.09, respectively. *AU_adjustedM_* of antipseudomonal beta-lactams at hospitals A and B could then be calculated as 100/(0.15/0.12) = 80 and 100/(0.09/0.12) = 133, respectively. The adjustments indicate that hospitals A and B would have utilized 80 and 133 DOT/1000 patient-days of antipseudomonal beta-lactams, respectively, if the proportion of *P. aeruginosa* isolates at both institutions were comparable to the overall network/regional average. Using 100 DOT/1000 patient-days of antipseudomonal beta-lactams may be justifiable in hospital A due to high microbiological burden, but seems excessive in hospital B.

Similar adjustments may be made for AU of anti-MRSA agents relative to the proportion of MRSA among all gram-positive isolates and AU of carbapenems based on proportion of ESBL-producing or ceftriaxone-resistant *E. coli*, *Klebsiella* species, and *Proteus mirabilis* (Table 2).

To our knowledge, these novel AU metrics in Section 4 have not been proposed in prior reviews of the literature. While their simple calculation is logical and represents a reasonable approach to AU interpretation, further research is warranted to validate these metrics in the clinical setting.

## 5. NHSN Antimicrobial Use and Resistance Module

The US Centers for Disease Control and Prevention (CDC) NHSN offers an antimicrobial use and resistance module with AU and AR options [80,81]. Facilities can participate in one or both options, but at this time, neither is required.

### 5.1. Antimicrobial Use (AU) Option

The AU option facilitates risk-adjusted inter- and intra-facility benchmarking of antimicrobial use [80]. Primarily, antimicrobial use is measured as antimicrobial DOT/1000 days-present. Antimicrobial use is aggregated by month for each patient care location and facility-wide. Antimicrobial use is also separated by the spectrum of the antimicrobials into 6 categories (Table 3). The data are then analyzed, and facilities receive a Standardized Antimicrobial Administration Ratio (SAAR) for each category and total antimicrobials in each patient care location and facility-wide. A SAAR of 1 indicates antimicrobial use is equivalent to referent populations. A SAAR greater than 1 that achieves statistical significance demonstrates excessive antimicrobial use, and a SAAR significantly lower than 1 may demonstrate underuse. SAAR still does not take into account quality of care (e.g., appropriateness of antimicrobial therapy). It does, however, attempt to control for institutional specifics, such as hospital size and complexity of patient population. The ability to look specifically at the different categories of antimicrobials is important, as ASPs are focused on decreasing broad-spectrum antimicrobials and increasing use of narrow-spectrum agents. This still constitutes a positive change for the institution, especially if SAAR for overall antimicrobials is equivalent to, or smaller than, 1, as it implies successful de-escalation from broad- to narrower-spectrum agents.

**Figure 1:**
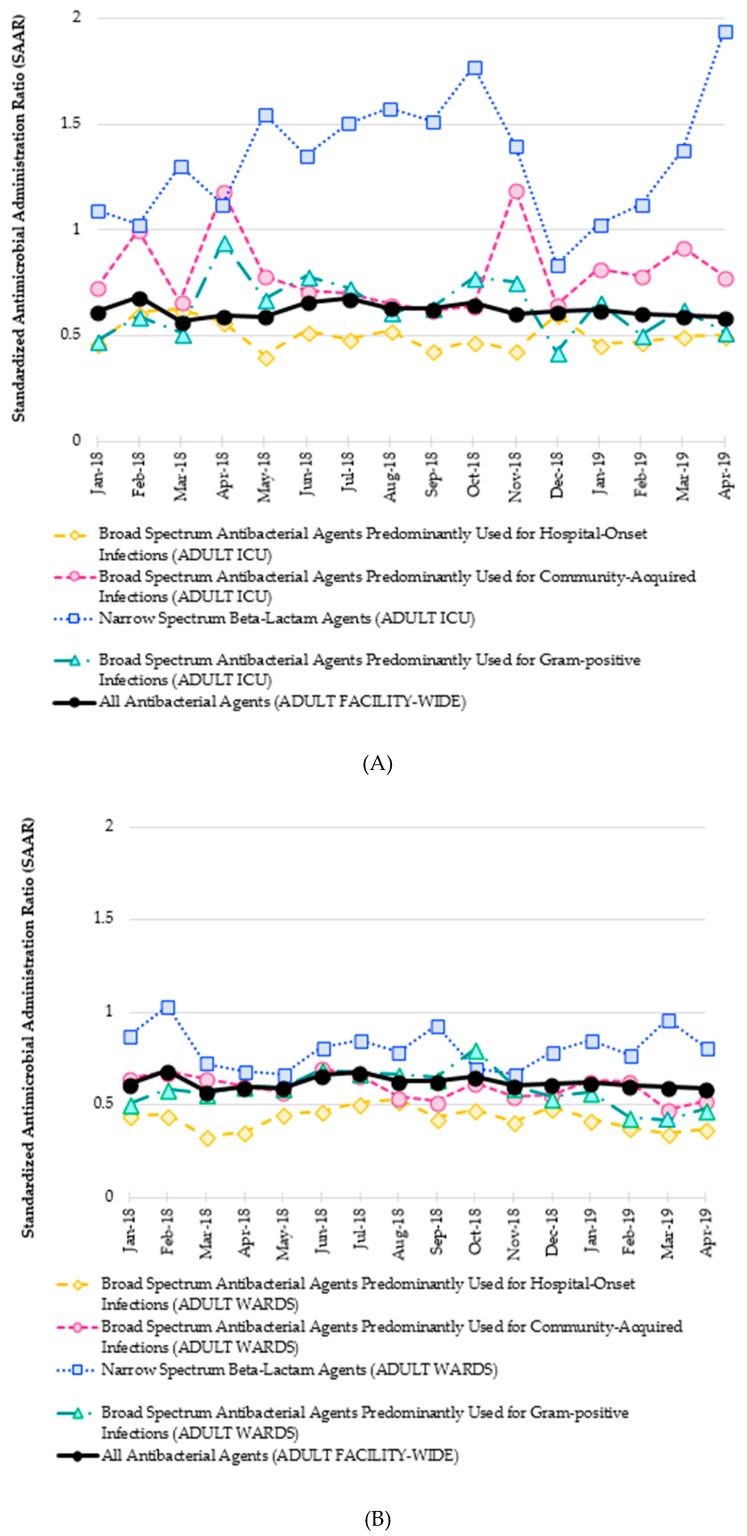
(**A**) Standardized Antimicrobial Administration Ratio (SAAR) report for all and select categories of antibacterial agents in adult intensive care units (ICUs) at a community-teaching hospital. (**B**) Standardized Antimicrobial Administration Ratio (SAAR) report for all and select categories of antibacterial agents at adult wards at a community-teaching hospital.

An example of such a comparison is provided in Figure 1. Such a line graph allows for a local ASP to evaluate their SAAR data over time. The monthly SAAR for all adult antibacterial agents facility-wide is in bold as an overall metric for antimicrobial use. Relevant subcategories are then superimposed on the same graph to allow for simple visual comparison and analysis by the ASP. Because subcategories for antibacterial agents are only available from NHSN for either adult intensive care units (ICUs) (Figure 1A) or adult wards (Figure 1B), the two line graphs are generated to assess antimicrobial use in each unit type. In this example, the adult facility-wide SAAR for overall antimicrobials remains below 1 for the entire time period. Broad-spectrum antimicrobials in adult ICUs are also consistently under 1, yet use of narrow-spectrum beta-lactams in adult ICUs is routinely above 1.0. This suggests that the observed use of agents such as penicillin G, ampicillin, nafcillin, and cefazolin was significantly greater than predicted by the NHSN model. Again, this antimicrobial use metric cannot assess quality of care. In order to distinguish whether this narrow spectrum beta-lactam use represents an appropriate de-escalation that an ASP can celebrate, versus an aggressive de-escalation which needs to be addressed by the ASP, this SAAR data would have to be coupled with quality of care data from the ICUs, e.g., appropriateness of therapy as previously discussed. In this way, SAAR data can reveal interesting nuances to guide local ASP assessments and subsequent initiatives. 

### 5.2. Antimicrobial Resistance (AR) Option

Facilities reporting to the AR option will receive a facility-wide antibiogram that can be stratified by source, time period, and specific antibiotics or organisms [80]. Participating facilities will also get a line list generated for all AR events including items such as date of birth, gender, specimen type, and organism. The benefits of this option for tracking the success of an ASP is similar to tracking MRSA, ESBLE, CRE, and resistance among *P. aeruginosa* and *A. baumannii* isolates. While short-term changes may be difficult to visualize, it may be possible to show long-term success. It also allows for benchmarking with other similar institutions. Both the AU and AR modules within the NHSN are promising ASP metrics with different potential uses.

## 6. Discussion

Historically, direct measurement of antimicrobial use was not feasible for most institutions; thus, surrogate metrics such as HO-CDI incidence and cost were derived. The assumption was that higher incidence rates of HO-CDI and infections due to MDR bacteria reflected excessive antimicrobial use of broad-spectrum agents at an institution. However, in the era of electronic medical records and progressive advancements of healthcare informatics, direct measurement of antimicrobial use has become a reality. CDC/NHSN module for reporting antimicrobial use and antimicrobial resistance provides the tools for direct measurements of ASP daily work and overall performance. Despite some initial hurdles, hospitals and ASPs are both determined to make this breakthrough advancement in the field of antimicrobial stewardship by improving ASP metrics. Adjustments of antimicrobial use by quality of care and institutional microbiological burden will evolve with time. Practical adjustment formulas, which demonstrate validity and generalizability across a broad mix of hospital types and geographical locations, will prove most useful.

ASP metrics may be classified into direct measures of ASP performance and global metrics for overall healthcare system evaluation. Antimicrobial use of broad-spectrum agents represents the primary direct ASP metric. Secondary direct metrics include incidence rate of CRE and antimicrobial resistance of predominantly hospital-onset pathogens, such as *P. aeruginosa* and *A. baumannii* (Table 4).

In addition to these direct metrics, global metrics, including rates of CDI, MRSA, ESBLE, as well as cost of healthcare, should continue to be used in order to evaluate overall performance of healthcare systems (Table 5).

## 7. Conclusions

In this new era of antimicrobial stewardship, direct measurement of ASP performance is feasible and preferable. Antimicrobial use within an institution represents the most direct and specific antimicrobial stewardship metric for hospital-based ASPs. Antimicrobial resistance of predominantly hospital-onset bacteria, such as *P. aeruginosa* and *A. baumannii,* represents a secondary antimicrobial stewardship metric. Participation of US hospitals in currently available CDC/NHSN modules for antimicrobial use and resistance is highly encouraged and represents a valuable step to improve antimicrobial stewardship at the national level. Novel stewardship metrics presented in this review allow adjustment of antimicrobial use by microbiological burden and quality of care as measured by appropriateness of empirical antimicrobial therapy at each institution. This enhances the antimicrobial stewardship mission in improving both the quantity and quality of patient care. 

## Figures and Tables

**Table 1:** Proposed novel metrics for adjustment of antimicrobial use by quality of care.

Adjusted AU	Formula
APBL	$\frac{AU_{APBL}}{\begin{array}{c} Proportion\;of\;patients\;with\;gram–negative\;BSI\;or\;sepsis\;\\ receiving\;appropriate\;empirical\;antimicrobial\;therapy \end{array}}$
Carbapenems	$\frac{AU_{Carbapenems}}{\begin{array}{c} Proportion\;of\;patients\;with\;gram–negative\;BSI\;or\;sepsis\;\\ receiving\;appropriate\;empirical\;antimicrobial\;therapy \end{array}}$
Anti-MRSA agents	$\frac{AU_{Anti-MRSA\;agents}}{\begin{array}{c} Proportion\;of\;patients\;with\;gram–positive\;BSI\;or\;sepsis\;\\ receiving\;appropriate\;empirical\;antimicrobial\;therapy \end{array}}$
Anti-VRE agents	$\frac{AU_{Anti-VRE\;agents}}{\begin{array}{c} Proportion\;of\;patients\;with\;gram–positive\;BSI\;or\;sepsis\;\\ receiving\;appropriate\;empirical\;antimicrobial\;therapy \end{array}}$

Note: AU: antimicrobial use; APBL: antipseudomonal beta-lactams; MRSA: methicillin-resistant *Staphylococcus aureus*; VRE: vancomycin-resistant *Enterococcus* species; BSI: bloodstream infections.

**Table 2:** Proposed novel metrics for adjustment of antimicrobial use by microbiological burden at each healthcare facility.

Adjusted AU	Formula
APBL	$\frac{AU_{APBL}}{\left(\frac{Incidence\;of\;P.\;\;aeruginosa\;at\;local\;institution}{Average\;overall\;incidence\;of\;P.\;\;aeruginosa\;in\;network}\right)}$
Carbapenems	$\frac{AU_{Carbapenems}}{{\left(\frac{Incidence\;of\;ESBLE\;at\;local\;institution}{Average\;overall\;incidence\;of\;ESBLE\;in\;network}\right)}^{*}}$
Anti-MRSA agents	$\frac{AU_{Anti-MRSA\;agents}}{\left(\frac{Incidence\;of\;MRSA\;at\;local\;institution}{Average\;overall\;incidence\;of\;MRSA\;in\;network}\right)}$
Anti-VRE agents	$\frac{AU_{Anti-VRE\;agents}}{\left(\frac{Incidence\;of\;VRE\;at\;local\;institution}{Average\;overall\;incidence\;of\;VRE\;in\;network}\right)}$

Note: AU: antimicrobial use; APBL: antipseudomonal beta-lactams; ESBLE: extended-spectrum beta-lactamase-producing *Enterobacteriaceae*; MRSA: methicillin-resistant *Staphylococcus aureus*; VRE: vancomycin-resistant *Enterococcus* species. * If microbiology laboratories in one or more hospitals in the network do not perform the ESBL screening test, then the incidence of ceftriaxone-resistant *Enterobacteriaceae* may be used alternatively to calculate adjusted carbapenem utilization in all hospitals in the network.

**Table 3:** Centers for Disease Control and Prevention National Healthcare and Safety Network antimicrobial use module categories.

Category	Commonly Used Antimicrobials
Broad-spectrum agents predominantly used for hospital-onset infections	Piperacillin/tazobactam, ceftazidime, cefepime, meropenem, imipenem/cilastatin, aztreonam, gentamicin, tobramycin
Broad-spectrum agents predominantly used for community-acquired infections	Ceftriaxone, cefotaxime, cefuroxime, cefdinir, ertapenem, ciprofloxacin, levofloxacin, moxifloxacin
Agents predominantly used for resistant gram-positive infections	Vancomycin, daptomycin, linezolid, ceftaroline
Narrow-spectrum beta-lactam agents	Penicillin G, ampicillin, amoxicillin, ampicillin/sulbactam, amoxicillin/clavulanate, nafcillin, dicloxacillin, cefazolin, cephalexin, cefoxitin
Agents posing the highest risk for *C. difficile* infection	Clindamycin, cefepime, ceftriaxone, cefdinir, ciprofloxacin, levofloxacin, moxifloxacin
Antifungal agents predominantly used for invasive candidiasis	Fluconazole, voriconazole, posaconazole, caspofungin, micafungin, anidulafungin

**Table 4:** Direct antimicrobial stewardship metrics.

ASP Metrics	Description
Antimicrobial use of broad-spectrum agents: Antipseudomonal beta-lactams Carbapenems Anti-MRSA agents Anti-VRE agents	Most direct measure of ASP performance Evaluates effectiveness of ASP interventions (e.g., syndrome-specific, prospective audit and feedback, de-escalation of therapy) Measures both empirical and definitive therapy Adjustments by quantity (facility size, patient population, or microbiological burden) and quality (appropriateness of therapy) at each healthcare facility are possible
Antimicrobial resistance of predominantly hospital-onset bacteria: Pseudomonas aeruginosa Acinetobacter baumannii	Antimicrobial resistance of hospital-onset bacteria is associated with use of broad-spectrum antimicrobials at each institution Antimicrobial resistance may also be influenced by referrals, especially at tertiary care centers Patient-to-patient transmission of MDR bacteria may be reduced by effective infection prevention and control methods
Incidence rate of CRE	Excessive use of carbapenems and other broad-spectrum antimicrobials increases risk of CRE infections or colonization CRE rates may be influenced by transfers from other hospitals or skilled nursing facilities Infection prevention and control programs are essential for reducing transmission of CRE in healthcare facilities

Note: ASP: antimicrobial stewardship programs; MRSA: methicillin-resistant *Staphylococcus aureus*; VRE: vancomycin-resistant *Enterococcus* species; MDR: multi-drug resistant; CRE: carbapenem-resistant *Enterobacteriaceae*.

**Table 5:** Global metrics of overall healthcare system performance.

Global Metrics	Description
Incidence rate of hospital-onset *Clostridioides difficile* infection	Indirect assessment of quantity and spectrum of AU in healthcare facilities Tool for evaluation of IPCPs, clinical decision support programs, laboratory, and diagnostic stewardship
Incidence rate of ESBLE infections or colonization	ESBLE predominantly cause community-onset infections in North America and Europe Better metric for ambulatory ASPs and IPCPs than inpatient ASPs Rates are influenced by prior colonization
Incidence rate of MRSA infections or colonization	MRSA has emerged as community-onset bacteria as well Association between MRSA and AU is not very clear Measures performance of IPCPs more than ASPs
Sepsis or bloodstream infection case-fatality rate	Evaluates clinical, diagnostic, and interventional critical care skills; clinical decision support programs; and laboratory diagnostics, including microbiology ASPs may influence only one of many variables that determine outcome, that is, empirical antimicrobial therapy through institutional management guidelines and other interventions
Cost of healthcare	Antimicrobial cost is a fraction of total healthcare cost ASPs may indirectly contribute to reduction in healthcare cost by reducing length of hospital stay through selection of appropriate empirical antimicrobial therapy and reducing risk of antimicrobial adverse events such as acute kidney injury and *C. difficile* infection

Note: ASP: antimicrobial stewardship programs; AU: antimicrobial utilization; ESBLE: extended-spectrum beta-lactamase-producing *Enterobacteriaceae*; IPCP: infection prevention and control program.

## Abbreviations

ASP = antimicrobial stewardship program; AR = antimicrobial resistance; AU = antimicrobial use; CDC = Centers for Disease Control and Prevention; CDI = *Clostridioides difficile* infection; CRE = carbapenem-resistant Enterobacteriaceae; DDD = defined daily dose; DOT = days of therapy; ESBLE = extended-spectrum beta-lactamase-producing Enterobacteriaceae; HO-CDI = hospital-onset *Clostridioides difficile* infection; ICU = intensive care unit; MDR = multidrug-resistant; MRSA = methicillin-resistant *Staphylococcus aureus*; NHSN = National Healthcare Safety Network; SAAR=Standardized Antimicrobial Administration Ratio
